# Evaluating the performance characteristics of five lateral flow assays for the detection of the SARS-CoV-2 nucleocapsid antigen

**DOI:** 10.1038/s41598-022-12805-1

**Published:** 2022-05-25

**Authors:** J. Heskin, S. J. C. Pallett, A. Al-Hindawi, G. W. Davies, M. Rayment, N. Mughal, P. Randell, R. Jones, L. S. P. Moore

**Affiliations:** 1 Chelsea and Westminster NHS Foundation Trust, 369 Fulham Road, London, SW10 9NH UK; 2 Centre of Defence Pathology, Royal Centre for Defence Medicine, Queen Elizabeth Hospital Birmingham, Mindelsohn Way, Edgbaston, Birmingham, B15 2WB UK; 3 North West London Pathology, Imperial College Healthcare NHS Trust, Fulham Palace Road, London, W6 8RF UK; 4 NIHR Health Protection Research Unit in Healthcare Associated Infections & Antimicrobial Resistance, Imperial College London, Du Cane Road, London, UK

**Keywords:** Infectious diseases, Viral infection

## Abstract

In response to the COVID-19 pandemic, lateral flow assays (LFAs) for the detection of SARS-CoV-2 antigen have been proposed as a complementary option to the more costly and time consuming reverse-transcriptase polymerase chain reaction (RT-PCR). We assessed five commercially available SARS-CoV-2 antigen detecting LFAs (ASSUT EUROPE (Rome, Italy), Besthree (Taizhou, China), Encode (Zhuhai, China), Fortress (Antrim UK), and Hughes Medical (Buckinghamshire, UK), using samples collected from hospitalised individuals with COVID-19 and compared these results against established RT-PCR assays with the aim of estimating test performance characteristics. We performed a diagnostic accuracy study of the five LFAs on 110 inpatients with confirmed COVID-19 and 75 COVID-19 negative control participants. Assay evaluation was performed using a modified version of each manufacturer’s protocol allowing for parallel testing of a single sample on multiple assays. Additional variables were studied including infection acquisition, oxygenation requirements at time of swabbing, and patient outcomes. The 110 patients were 48% (53) female, with mean age 67 years (range 26–100 years), and 77% (85) cases were community onset SARS-CoV-2. Across the five assays, sensitivity ranged from 64 (95% CI 53–73) to 76% (95% CI 65–85); Fortress performed best with sensitivity of 76% (95% CI 65–85). Specificity was high across all assays with 4/5 LFAs achieving 100%. LFA sensitivity was not dependant on RT-PCR cycle thresholds. SARS-CoV-2 antigen detecting LFAs may complement RT-PCR testing to facilitate early diagnosis and provide community testing strategies for identification of patients with COVID-19, however we find suboptimal test performance characteristics across a range of commercially available manufacturers, below WHO and MHRA pre-set sensitivity performance thresholds. With such variation in sensitivity between LFAs and PCR testing and between assay brands, we advise caution in the deployment of LFAs outside of environments with clinical oversight.

## Introduction

In response to the COVID-19 pandemic, testing capacity for clinical diagnostics and for screening have been stretched at a global level. Increasing demand has meant that novel testing platforms and protocols are required, with an expectation that they will play a role in both individual healthcare and community testing programmes, national population studies, and international epidemiological guidance.

Reverse transcriptase polymerase chain reaction (RT-PCR) is considered the gold standard test for the detection of SARS-CoV-2 infection, but has a significant processing time requiring specific laboratory infrastructure^1^. Whilst some advances have been made in laboratory-free RT-PCR platforms, these are still not in widespread use in large scale community testing^2,3^. A faster and more portable alternative is required and lateral flow assays (LFAs) for the detection of SARS-COV-2 antigen have been proposed as a complementary option^4^. During the rapid development of SARS-CoV-2 antibody lateral flow assays, significant inter-manufacturer variation was seen, with some performing particularly poorly^5–7^. If antigen LFAs are to be used to complement PCR testing, evaluation for any similar inter-manufacturer variation must be analysed.

Whilst laboratory based assessment of these rapid antigen LFA assays has occurred, evaluation of assays in the setting of intended use with both sample and operator skill-sets who are representative of clinical and community testing sites remains unstudied. In the UK, testing has focused on one specific manufacturer, procured at a national level^8,9^, whilst competitor assays also remain unstudied. In this analysis we assessed five commercially available SARS-CoV-2 antigen LFAs using samples collected from hospitalised individuals with COVID-19 and compared these results against established RT-PCR assays with the aim of estimating test performance characteristics.

## Methods

### Study design

We performed a prospective diagnostic accuracy study, independent of manufacturers, to evaluate five commercially available SARS-CoV-2 antigen point-of-care lateral flow assays between November 2020 and February 2021. This was a single centre study, based in a large urban hospital, comprising 110 in-patients and 75 SARS-CoV-2 negative controls. Clinicians identified participants, collected clinical details including the oxygen requirements and infection acquisition source of each patient, and performed nasopharyngeal swabs for inoculation of the lateral flow assays. Follow-up data on patient survival was also collected at the end of the study.

SARS-CoV-2 antigen LFAs were identified through our procurement team as viable alternatives to the nationally procured LFA^8,9^. Initial assessment was performed with all assays required to meet the following criteria: a cassette-based design with visual read-out result in less than 20 min, and manufacturer reported sensitivity of > 95% and specificity of > 95%, availability for purchase in the United Kingdom, and accreditation via a CE mark. Five assays were selected for in-depth evaluation; ASSUT EUROPE (Rome, Italy), Besthree (Taizhou, China), Encode (Zhuhai, China), Fortress (Antrim UK), and Hughes Medical (Buckinghamshire, UK). The target of all selected assays was the SARS-CoV-2 nucleocapsid protein antigen. All evaluated assays were approved for nasopharyngeal sampling. As all assays were CE marked, this service evaluation was undertaken as a validation of these in vitro diagnostic devices in line with the UK Standards for Microbiological Investigation structure^10^. The service evaluation was registered with the hospital Clinical Governance Department, and all participants provided informed consent for use of their samples for the purpose of assay validation.

### SARS-CoV-2 positive cohort

Patients confirmed to have COVID-19 were identified via a centralised daily report of all SARS-CoV-2 results performed in the previous 24 h within our Trust. Patients’ electronic health records were examined to assess for suitability according to the inclusion criteria; current inpatient at Chelsea and Westminster Hospital NHS Foundation Trust, positive RT-PCR test for SARS-CoV-2 within the previous 72 h, symptom onset < 14 days (in those with symptoms), ability to provide informed consent. Patients were excluded if; age < 18 years old, unable to consent due to pre-existing medical condition or acute alteration in an individual’s condition, symptom onset > 14 days, PCR positive for SARS-CoV-2 > 72 h, asymptomatic without a negative swab within the previous 14 days. The laboratory serving the hospital network used a variety of PCR platforms to derive COVID-19 status, including AusDiagnostics (Mascot, Australia), Roche Cobas 6600 (Roche Molecular Systems, New Jersey, US), and Abbott RealTime (Illinois, US) platforms.

### SARS-CoV-2 negative control cohort

Negative controls were recruited from hospital staff, and students from Imperial College School of Medicine. In line with NHS England guided testing of healthcare professionals, all individuals recruited undertook routine twice weekly LFA testing using the aforementioned nationally procured Innova assay. Additionally, all recruited members from the Haematology, Oncology and HIV teams were undergoing weekly PCR testing in keeping with local policy on health care professionals working with immunocompromised patients. All individuals recruited as negative controls were asymptomatic at time of testing and had a negative Innova LFA or RT-PCR within the previous 72 h.

### Evaluation protocol

Assay evaluation was performed using a modified version of each manufacturer’s protocol. This allowed for parallel testing of a single sample (nasopharyngeal swab) across the five assays. These modifications included an increase in the quantity of extraction buffer used from 300 to 600 µl and the use of a single extraction solution with sodium azide 0.09% preservative. This protocol deviation was evaluated using five positive and five negative *ACON Biotech5* (Hangzhou, China) SARS-CoV-2 positive control swabs on each of the five assays and compared to the original manufacturers protocol. There was 100% agreement between the modified and the original protocol in positive and negative control cases.

The nasopharyngeal swabbing procedure followed manufacturer protocol and all samples were taken by a trained member of the research team to ensure consistent sampling technique. Samples were stored in refrigerated temperatures (2–4 degrees centrigrade). All assays with a visible control line were deemed valid. Chromatographic results on each assay were read by a single member of staff trained in the use of lateral flow assays including interpretation of results. In the case of an inconclusive result, a second member of the research team was consulted to reach a final decision.

### Statistical analysis

Each LFA result was compared to the RT-PCR result. Cycle threshold values were not available for all RT-PCR samples and thus absent results were imputed via multiple imputations using chained equations^11^. This technique facilitates the use of missing data whilst propagating the uncertainty in estimating missing data, thus minimising selection bias. The dataset is imputed 20 times, using differing random seeds, resulting in 20 different possible values for each missing value. Each imputed dataset’s performance is then aggregated, and the spread is calculated and displayed for the appropriate metric.

To understand the effects of the RT-PCR cycle threshold (CT) and temporal delay between swabbing on the accuracy of LFAs, receiver operating characteristic (ROC) curves were constructed for each assay.

### Ethical approval

This evaluation was commissioned as a service evaluation by the COVID Testing Committee of Chelsea & Westminster NHS Foundation Trust, on 20 October 2020. The study was reviewed by the Chelsea & Westminster NHS Foundation Trust Research and Development Office and deemed a verification of a CE marked in vitro diagnostic test; therefore, informed consent was required to participate but this was not required to be in written format. Aggregated data was analysed under the Health Service Control of Patient Information Regulations (2002) general notice that patient data for a COVID-19 purposes may be used for research as stated by the UK Secretary of State for Health and Social Care. The study was conducted in accordance with relevant guidelines and regulations including the Declaration of Helsinki.

## Results

### Patient/negative control cohort

As detailed, the SARS-CoV-2 positive cohort consisted of 110 inpatients, with each assay tested against 75 of these individuals. The cohort was almost evenly split by gender, 52% (57) male and 48% (53) female and ages ranged from 26 to 100 years old, with a mean age of 67 years. Almost half (46%, 51) of the cohort were in the higher risk age category (> 70 years old). Community acquisition of SARS-CoV-2 accounted for 77% (85) of our positive cohort with the remaining 23% (25) resulting from nosocomial transmission (see Table 1).

**Table 1:** Patient cohort—demographics and data.

Gender	No.	Age	No.	Acquisition source	No.	Oxygen delivery method	No.	Outcome at data collection completion	No.
Male	57	25–34	6	Community	85	Room air	55	Discharged	67
Female	53	35–44	6	Hospital	25	Nasal O2	28	Inpatient	17
		45–54	21			Non rebreathe/Venturi	14	Died	26
		55–64	13			NIV	13		
		65–74	21						
		75–84	21						
		85–94	20						
		95–100	2						

Hypoxia requiring supplemental oxygen was a common symptomatic feature. At the point of LFA sampling, 50% (55) of patients were not requiring supplemental oxygen, 38% (42) were receiving oxygen via nasal cannula or by non-rebreathe and Venturi face masks, while 12% (13) were receiving positive pressure non-invasive ventilation. At the completion of data collection 61% (67) of the cohort had been discharged from hospital, 15% (17) remained in hospital undergoing rehabilitation, and 24% (26) of the cohort had died (see Table 1).

In total, 75 negative control individuals were enrolled in the validation process. The cohort was 51% (38) male and 49% (37) female with an age range of 21–64 years (mean: 34 years of age) (see Table 2).

**Table 2:** Negative control cohort—demographics.

Gender	No.	Age	No.
Male	38	25–34	20
Female	37	35–44	23
		45–54	18
		55–64	12
		65–74	2
		75–84	0
		85–94	0
		95–100	0

### LFA test performance characteristics

The sensitivity of the LFAs ranged from 64% (95% CI 53–73) to 76% (95% CI 65–85). Of the assays tested, Fortress performed best with a sensitivity of 76% (95% CI 65–85). Specificity based on testing SARS-CoV-2 volunteers was 100% (95% CI 95–100) for four of the LFA’s), except for Encode with 99% (95% CI 92–100) (see Table 3, Supplementary Receiver Operating Curve Figures).

**Table 3:** LFA performance characteristics.

Brand	Sensitivity (%)	95% CI	Specificity (%)	95% CI
Fortress	76	(65–85)	100	(95–100)
Hughes	72	(61–81)	100	(95–100)
Encode	71	(60–80)	99	(92–100)
Besthree	68	(57–77)	100	(95–100)
Assut	64	(53–73)	100	(95–100)

### Impact on time from RT-PCR to LFA test performance characteristics

Time from RT-PCR swab to LFA swab was examined with 46% (51) of participants sampled within 24 h, 34% (38) within 48 h and 20% (21) within 72 h of a positive RT-PCR test. No patients were sampled at greater than 72 h of a confirmed RT-PCR result. None of the LFAs showed significant dependence on the time between RT-PCR tests and LFA swab.

### Correlation of RT-PCR cycle threshold and LFA test performance characteristics

**Figure 1:**
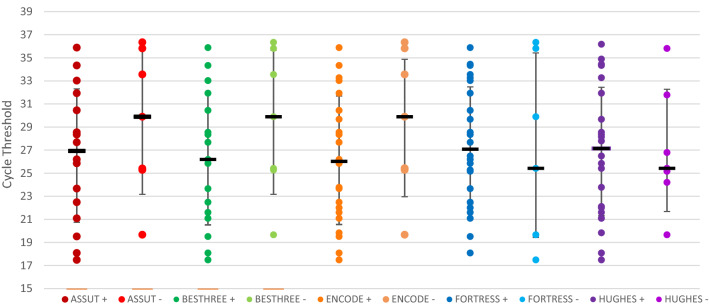
Correlation between SARS-CoV-2 antigen positive and negative results and corresponding RT-PCR cycle threshold across five commercially available lateral flow devices, London, 2021.

The sensitivity of each LFA was explored as a surrogate marker of the RT-PCR cycle threshold using ROC curves. No particular test demonstrated reliance on a specific RT-PCR cycle threshold to achieve positivity (Fig. 1). Instead, across all five assays there were relatively constant median cycle thresholds (of around 27) among those LFDs that were positive, and among those LFDs that were negative, suggesting that likelihood of nasopharyngeal antigen detection is independent of the PCR cycle threshold.

## Discussion

Our study finds variation between 5 major manufacturer’s SARS-CoV-2 antigen lateral flow devices, in particular there is marked variation in sensitivity when compared to RT-PCR. While other studies examining antigen detecting LFAs have utilised recombinant nucleoproteins, viral culture samples and stored respiratory samples, our study demonstrates significant inter-manufacturer sensitivity (and particularly variability in time-from-RT-PCR-positive) using clinical in situ methodology.

The range of manufacturer reported sensitivity values (78% to 99%) differed substantially from the sensitivity determined in our evaluation (64% to 76%, 95% CI (53–85)), highlighting the importance of independent assessment of product performance in real clinical settings with representative clinician-user skill sets. Our findings are corroborated by results from a PHE Porton Down study which showed a drop in sensitivity of the UK government procured Innova antigen LFA^8,12^ when real-world samples, and operators, are used. The between-assay variation in sensitivity we demonstrated also suggests that interchangeability between different manufacturers could significantly impact accuracy in testing.

Based on our evaluation, none of the five assays meet the WHO and MHRA’s priority target product profiles for COVID‐19 diagnostics, where acceptable sensitivity is deemed ≥ 80% and specificity ≥ 97%. Thus, none can be considered as a replacement for laboratory-based RT-PCR^13,14^. They may, however, represent a useful diagnostic adjunct in the current SARS-CoV-2 pandemic, its potential future endemic presence, and in regions where RT-PCR testing remains limited. With any highly infectious condition, time sensitive decisions regarding patient placement in hospital are required and laboratory-based RT-PCR cannot always meet this demand. SARS-CoV-2 antigen LFAs may be best suited to situations where confirmatory RT-PCR testing can be performed once the patient is in a more secure environment.

There are several limitations to our study including deviation from the manufacturer protocol, although our verification with set laboratory controls indicated this had no effect. Secondly, we undertook non-contemporaneous RT-PCR and LFA sampling. This decision was pragmatic, as unlike antibody LFAs which can be verified using excess/waste serum, antigen LFAs require nasal or nasopharyngeal samples for which there is no excess. Tolerability of repeated nasopharyngeal swabs impacts the ability to perform head-to-head comparisons on individual patients. Variation in quantity and quality of sampling compared to the initial RT-PCR swabs may impact on the reported assay sensitivity, but we mitigated this by using clinicians experienced in nasopharyngeal swabbing, to obtain all samples. There remains a possibility that an individual’s antigen positive status may have waned after their initial RT-PCR positive result, but our analysis of the impact of time-between-sampling demonstrated minimal impact of this variable, implying that this is unlikely to have had a significant impact on the LFA poor sensitivity. Finally, RT-PCR testing was performed on a number validated platforms, and CT was not available to enable comparison for all assays (some were tested on the dnaNudge platform)^2^. Reassuringly, our investigation of correlation between RT-PCR CT and LFA positivity demonstrated little effect here, yet in other centres there has been a demonstrable correlation between the likelihood of LFD positivity and RT-PCR cycle thresholds^13^. We would caution relating this to infectivity however, given yet other groups have demonstrated that RT-PCR cyle thresholds do not necessarily correlate to infectivity^14^.

In conclusion, while SARS-CoV-2 antigen LFAs may complement RT-PCR testing to facilitate early diagnosis and provide community testing strategies for identification of patients with COVID-19, we find suboptimal test performance characteristics across a range of commercially available manufacturers, below pre-set sensitivity performance thresholds^15,16^. With limited access to healthcare facilities due to pandemic restrictions, the ability for patients to perform home sampling greatly reduces the demand on hospital services and the need for unecessary travel to, and footfall within, clinical settings. Demand for a rapid, self-administered point of care diagnostic tool remains paramount, however with such variation in sensitivity between LFAs and RT-PCR testing and between assay brands, we advise caution in the deployment of LFAs outside of environments with clinical oversight.

## Supplementary Information

Supplementary Figure S1.

## Data Availability

The data analysed during the current study and further details on the assays are available from the corresponding author (LSPM; l.moore@imperial.ac.uk) on reasonable request, as long as this meets local ethical and research governance criteria.
