# Supplementary material for: Evaluating the performance characteristics of five lateral flow assays for the detection of the SARS-CoV-2 nucleocapsid antigen
Source: Sci Rep. 2022 May 25;12:8811. doi: 10.1038/s41598-022-12805-1 (PMC9130972; doi:10.1038/s41598-022-12805-1)
Supplement: Supplementary file 1 — Supplementary Figure S1. [file 41598_2022_12805_MOESM1_ESM.docx]

**Supplementary Figure.**

**Receiver operating curves for five commercial SARS-CoV-2 antigen lateral flow devices against RT-PCR gold standard, 110 patients, London, 2021.**

ASSUT

Besthree

**
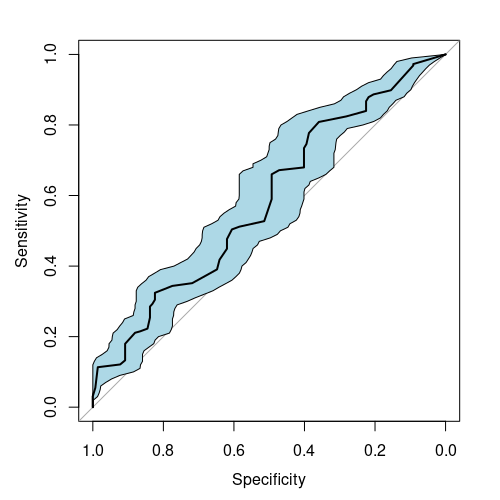

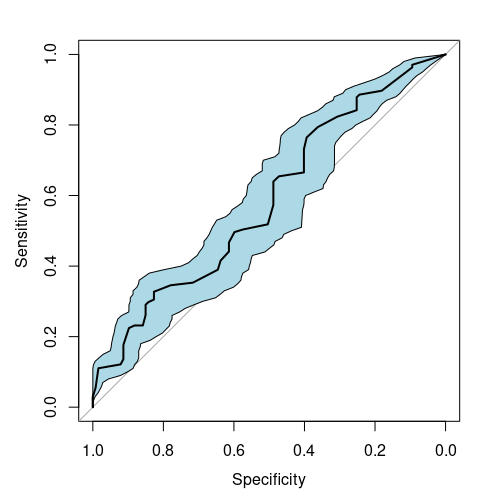
**

Fortress

Encode

**
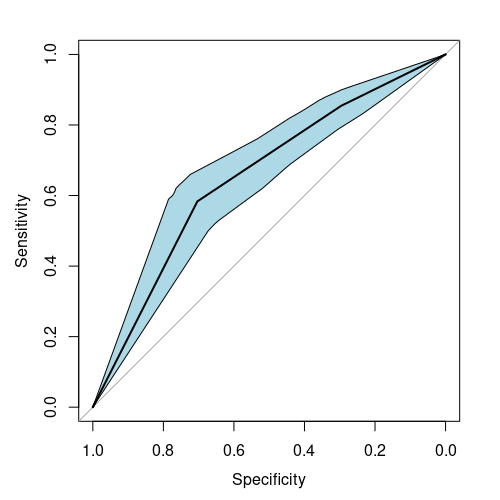

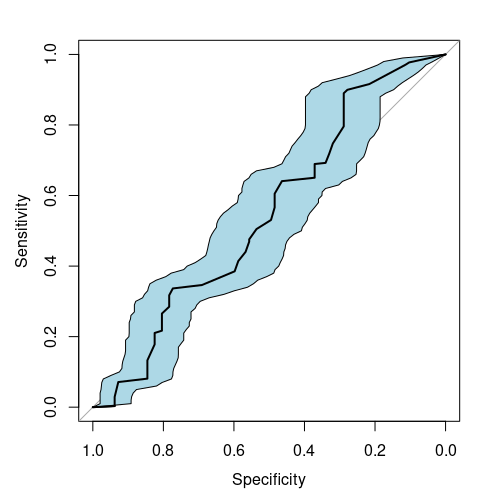
**

Hughes

**
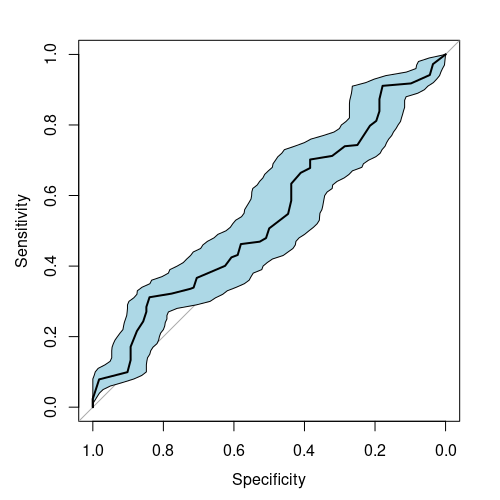
**
